# Emerging roles of keratinocytes in nociceptive transduction and regulation

**DOI:** 10.3389/fnmol.2022.982202

**Published:** 2022-09-09

**Authors:** Xiaohan Xu, Catherine Yu, Li Xu, Jijun Xu

**Affiliations:** ^1^Department of Anesthesiology, Chinese Academy of Medical Sciences & Peking Union Medical College Hospital, Beijing, China; ^2^Department of Pain Management, Anesthesiology Institute, Cleveland, OH, United States; ^3^Department of Inflammation and Immunity, Lerner Research Institute, Cleveland, OH, United States; ^4^Cleveland Clinic, Case Western Reserve University, Cleveland, OH, United States

**Keywords:** keratinocyte, free nerve ending, peripheral sensitization, neurotransmitter, neuropeptide, neuroinflammation

## Abstract

Keratinocytes are the predominant block-building cells in the epidermis. Emerging evidence has elucidated the roles of keratinocytes in a wide range of pathophysiological processes including cutaneous nociception, pruritus, and inflammation. Intraepidermal free nerve endings are entirely enwrapped within the gutters of keratinocyte cytoplasm and form *en passant* synaptic-like contacts with keratinocytes. Keratinocytes can detect thermal, mechanical, and chemical stimuli through transient receptor potential ion channels and other sensory receptors. The activated keratinocytes elicit calcium influx and release ATP, which binds to P2 receptors on free nerve endings and excites sensory neurons. This process is modulated by the endogenous opioid system and endothelin. Keratinocytes also express neurotransmitter receptors of adrenaline, acetylcholine, glutamate, and γ-aminobutyric acid, which are involved in regulating the activation and migration, of keratinocytes. Furthermore, keratinocytes serve as both sources and targets of neurotrophic factors, pro-inflammatory cytokines, and neuropeptides. The autocrine and/or paracrine mechanisms of these mediators create a bidirectional feedback loop that amplifies neuroinflammation and contributes to peripheral sensitization.

## Introduction

Keratinocytes (KCs) are the predominant cells that make up about 95% of cells in the epidermis. The epidermis is divided into four layers: stratum basale, spinosum, granulosum and corneum (Roger et al., 2019). KCs in stratum basale are mitotically active cells (Houben et al., 2007; Roger et al., 2019). Their proliferation is responsible for epidermis turnover (Houben et al., 2007; Roger et al., 2019). As KCs migrate superficially, they lose replicative potential and undergo differentiation (Houben et al., 2007). During this process, they deposit keratin, which adds to skin mechanical strength (Vidal Yucha et al., 2019). In the stratum corneum, KCs lose nuclei, release lipids, and terminally differentiate into squamous corneocytes (Roger et al., 2019). Keratin-filled corneocytes and densely packed intercellular lipids combine to form the skin barrier (Roger et al., 2019).

The proliferation, differentiation, and migration of KCs are crucial for the maintenance of skin homeostasis (Gallegos-Alcala et al., 2021). However, KCs are not just the structural backbone of the epidermis. Mounting evidence has showed the wide versatility of KCs. KCs have emerged as a key player in a wide range of pathophysiological processes including cutaneous nociception, pruritus, and inflammation.

In this review, we aim to present available evidence on the roles of KCs in nociceptive transduction and regulation. Nociceptive transduction is defined as peripheral terminals of nociceptive C fibers and A-delta (Aδ) fibers depolarized (to generate action potential) and activated by noxious mechanical, thermal, or chemical stimuli. KCs express various sensory receptors that can convert noxious stimuli into an action potential to get activated (Talagas et al., 2020a). The activated KCs contribute to nociceptive transduction by triggering action potentials in peripheral nociceptors in intraepidermal free nerve endings (FNEs) (Baumbauer et al., 2015; Moehring et al., 2018). We first elucidate the anatomical relationship and neurotransmission between KCs and intraepidermal nerve fibers. Next, we summarize the sensory and neurotransmitter receptors expressed on KCs, with a special focus on their contributions to nociceptive transduction. KCs can also be a source of cytokines, neurotrophic factors, neuropeptides, and neurotransmitters, which enhance their interaction with neighboring nerve fibers and immune cells (Vidal Yucha et al., 2019). In this way, KCs participate in nociceptive regulation. We also review the pivotal role of KCs in neuroinflammation and peripheral sensitization.

## Anatomical relationship between keratinocytes and intraepidermal free nerve endings

**FIGURE 1:**
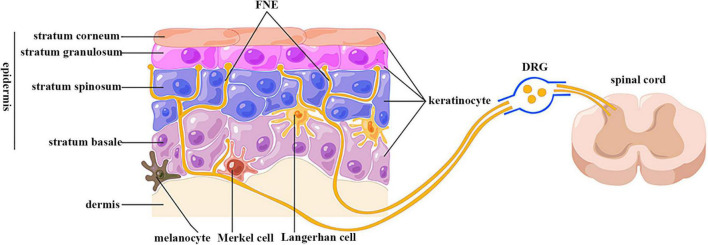
Anatomical relationship between keratinocytes and sensory nerve endings. FNE, free nerve ending; DRG, dorsal root ganglion. Epidermis is divided into four layers: stratum basale, spinosum, granulosum, and corneum. Keratinocytes are the predominant cells in epidermis. The epidermis is highly innervated with intraepidermal free nerve endings, which are enwrapped but also ensheathed by keratinocyte cytoplasm. Their cell bodies are located in dorsal root ganglion, and are centrally projected to spinal cord dorsal horn. (By Figdraw).

The epidermis is highly innervated with intraepidermal FNEs expressing peripheral nociceptors whose cell bodies are localized in dorsal root ganglion (DRG) and trigeminal ganglion (TG) (Figure 1) (Talagas et al., 2018). The central projections of these sensory neurons transduce nociception (Basbaum et al., 2009). Passing tortuously between KCs, FNEs are able to detect mechanical, thermal, and chemical noxious stimuli, and are thus called “nociceptors” (Basbaum et al., 2009). Nociceptive FENs are generally divided into two categories: unmyelinated small-diameter C-fibers transducing slow and poorly localized pain, and thinly myelinated medium-diameter Aδ-fibers transducing fast and well localized pain (Basbaum et al., 2009).

### Wrapping of free nerve endings within keratinocyte cytoplasm

The utilization of microscopy and immunolabeling techniques has made it possible to investigate the anatomical relationship between FNEs and KCs. Electron microscopy data showed that FNEs are enwrapped within the gutters of KC cytoplasm over their entire circumference in biopsies of human glabrous and hairy skin (Chouchkov, 1974; Cauna, 1980; Hilliges et al., 1995; Talagas et al., 2020c). Immunostaining results indicated these FNEs were almost exclusively Aδ and C fibers in a KCs–sensory neuron coculture model (Talagas et al., 2020c). This neuroanatomical relation was also observed by the confocal laser scanning microscopy (Talagas et al., 2020b). Also in this coculture model, KCs stretch outgrowths that embrace FNEs (Talagas et al., 2020c). FNE ensheathment by KCs contributes to the morphogenesis and function of nociceptive sensory neurons in *Drosophila*; this evolutionarily conserved mechanism also exists in humans (Jiang et al., 2019; Talagas et al., 2020b).

#### *En passant* synaptic-like contact

Several studies provided ultrastructural and functional evidence on the presence of *en passant* synaptic-like contacts between KCs and FNEs. In human hairy skin, FNEs are closely apposed to the cell bodies or cilia of KCs without any specialized structures between their plasma membrane (Hilliges et al., 1995). This close contact was also visualized by the atomic force microscopy (AFM) in nanoscale in cocultured KCs and sensory nerve endings (Klusch et al., 2013). The adjacent KC membrane is slightly thickened, resembling a synaptic membrane (Chateau and Misery, 2004). However, using *in situ* correlative light electron microscopy (CLEM), Talagas et al. (2020c) did not observe membrane thickening. Instead, they identified presynaptic vesicle markers in a pearl necklace pattern in KC cytoplasm in the immediate vicinity of FNEs, but not at a distance from FNEs (Talagas et al., 2020c). The density of these vesicles is increased in skin biopsies from small fiber neuropathy (SFN) (Talagas et al., 2020c). A key molecule for exocytosis (syntaxin 1A) is also present in KC cytoplasmic gutters around FNEs, and is required for the communication with sensory neurons (Talagas et al., 2020c). Although the percentage of FNE enwrapped by KCs or forming a synaptic-like structure with KCs has not been reported, the integrated anatomical relation between KCs and FNEs forms the basis of connection and communication between them.

## Sensory and neurotransmitter receptors expressed on keratinocytes

From conventional point of view, intraepidermal FNEs are the exclusive cutaneous nociceptors (Talagas et al., 2020a). However, accumulating data have highlighted the role of KCs in detecting and transducing noxious stimuli through their expression of a variety of sensory and neurotransmitter receptors. These sensory receptors expressed by KCs also contribute to the maintenance of skin hemostasis. The *in vivo* evidence on the roles of individual ion channels/receptors expressed on KCs in nociception was summarized in Table 1.

**TABLE 1:** Summary of *in vivo* evidence on the roles of sensory and neurotransmitter receptors expressed on keratinocytes in nociceptive transduction and regulation.

Receptor	*In vivo* evidence	Potential role
TRPV1	*In vivo* K14-positive cell-specific activation induced neural activation in spinal cord dorsal horn and acute nociceptive behaviors. Enhanced expression on KCs in a rat model of CRPSI, and in skin biopsies from patients with Herpes Zoster, SFN, DPN, and nerve injury.	Transduction
TRPV3	*In vivo* K14-positive cell-specific overexpression induced augmented nociceptive behaviors only when TRPV1 was inhibited. *In vivo* global knockout did not induce changes in thermal thresholds (possibly due to the existence of redundant molecules). Enhanced expression on KCs in human painful breast tissues.	Regulation
TRPV4	*In vivo* K14-positive cell-specific knockout induced attenuated UVB-induced nociceptive behaviors.	Regulation
TRPA1	*In vivo* K14-positive cell-specific knockout induced attenuated mechanosensitivity. These effects might rely on Merkel cells, since mice KCs express little TRPA1.	Regulation
STIM1	*In vivo* K14-positive cell-specific (but not neuron-specific) knockout induced reduced warm-responding DRG neurons and altered temperature preference, but retained normal responsiveness to noxious heat.	Regulation
VGSC	Enhanced expression on KCs in skin biopsies from patients with CRPS, PHN and SFN	Regulation
α1-AR	Enhanced expression on KCs in rat models of CCI or burn. Enhanced expression on KCs in bilateral limbs in a rat model of CRPS I. Systematic, but not intraplantar administration of an antagonist attenuated nociceptive behaviors, suggesting a central, but not a peripheral mechanism.	Regulation
NMDAR	Enhanced expression and phosphorylation on KCs in a rat model of CRPS II	Regulation
AMPAR	Decreased expression on KCs in skin biopsies from patients with PHN	Regulation
GABAR	Enhanced expression on KCs in mice with formalin-evoked pain	Regulation

TRPV, transient receptor potential vanilloid; TRPA, transient receptor potential ankyrin; KC, keratinocyte; K14, keratin 14; CRPS, complex regional pain syndrome; SFN, small fiber neuropathy; DPN, diabetic peripheral neuropathy; PHN, post-herpetic neuralgia; CCI, chronic constriction injury; NMDAR, N-methyl-D-aspartate receptor; AMPAR, α-amino-3-hydroxy-5-methyl-4-isoxazolole propionate receptor; GABAR, γ-Aminobutyric acid receptor; UV, ultraviolet irradiation. K14-positive cells include basal and suprabasal KCs and Merkel cells.

### Transient receptor potential ion channel

**FIGURE 2:**
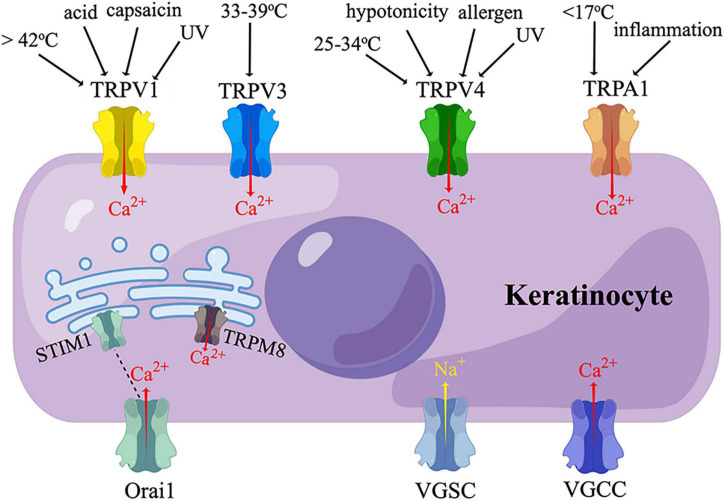
Sensory receptors expressed on keratinocytes. TRPV, transient receptor potential vanilloid; TRPA, transient receptor potential ankyrin; TRPM, transient receptor potential melastatin; VGSC, voltage-gated sodium channel; VGCC, voltage-gated calcium channel; UV, ultraviolet irradiation. Human keratinocytes express TRPV1, TRPV3, TRPV4, and TRPA1, which are activated by thermal, cold, chemical, or ultraviolet stimuli, and mediate a calcium influx. TRPM8 and STIM1 are temperature-sensitive endoplasmic reticulum transmembrane channels expressed in human keratinocytes. STIM1 is coupled to its plasma membrane subunit, Orail, which also mediate a calcium influx. VGSC and VGCC have also been found in human keratinocytes. (By Figdraw).

Transient receptor potential (TRP) proteins are non-selective ion channels permeable to Na+, Ca2+, and Mg2+ (Shirolkar and Mishra, 2022). TRPs are sensors of thermal, mechanical, and chemical stimuli, participating in nociception, pruritus, inflammation, and cell proliferation (Shirolkar and Mishra, 2022). Mammalian TRPs are classified into two main groups depending on their similarity to Drosophila TRPs (Shirolkar and Mishra, 2022). Group 1 includes TRP canonical (TRPC), TRP vanilloid (TRPV), TRP melastatin (TRPM), TRP ankyrin (TRPA), and TRPN (no mechanoreceptor potential C) (Shirolkar and Mishra, 2022). Among them, TRPV1, TRPV3, TRPV4, TRPA1, and TRPM8 have been found on human KCs, evoking a Ca^2+^ influx upon activation (Figure 2) (Inoue et al., 2002; Radtke et al., 2011; Bidaux et al., 2015; Luostarinen et al., 2021).

TRPV1 is activated by a temperature above 42°C, capsaicin, low pH, ultraviolet (UV) irradiation, or shear stress (Caterina et al., 1997; Inoue et al., 2002; Graham et al., 2013; Masumoto et al., 2013). TRPV1 is widely distributed in primary afferent neurons, and is also expressed in other tissues at a low level, including epidermal KCs (Lee and Caterina, 2005). Enhanced expression of TRPV1 on KCs was observed in a rat cast immobilization model of complex regional pain syndrome I (CRPS I) (Sekino et al., 2014). The expression of TRPV1 was also increased in the epidermal KCs of patients with Herpes Zoster, SFN, diabetic peripheral neuropathy (DPN), and nerve injury; with the expression level correlated with pain intensity (Facer et al., 2007; Wilder-Smith et al., 2007; Han et al., 2016). It is not clear whether the KCs selectively or broadly expressing TPRV1 are in direct contact with TRPV1-expressing or all the FNEs.

Keratin 14 (K14) is a marker of mitotically active cells in stratified squamous epithelia, including skin, hair follicle, tongue, mouth, esophagus, forestomach, and thymus (Vassar et al., 1989). By crossing mice with a target gene and mice with Cre under the control of the K14 promoter, investigators created transgenic mice that expressed the target gene predominantly in basal and suprabasal KCs along with other K14-expressing cells, such as Merkel cells (Hafner et al., 2004; Baumbauer et al., 2015; Moehring et al., 2018). In a mouse line exclusively expressing TRPV1 in K14-positive cells, a subcutaneous injection of capsaicin was sufficient to induce significant expression of c-fos, a marker of neural activation, in laminae I and II of the ipsilateral spinal cord dorsal horn and acute nociceptive behaviors (Pang et al., 2015). These findings indicated that selective stimulation of TRPV1 on KCs (in the absence of TRPV1 on nociceptors) is sufficient to trigger nociceptive transduction. Future *in vivo* KC-selective TRPV1 knockout studies are valuable to confirm the essential role of KCs TRPV1 in nociceptive transduction. Furthermore, KCs TRPV1 participates in the development of cutaneous inflammation. TRPV1 activation induced a production of pro-inflammatory mediators, including cyclooxygenase-2 (COX-2), prostaglandin E2 (PGE2), interleukin (IL)-8, IL-1β, IL-2, IL-4, and tumor necrosis factor (TNF)-α in KCs (Southall et al., 2003; Lee et al., 2011; Yoo et al., 2020). The expression of TRPV1 is upregulated by heat, UV irradiation, and blue light in human KCs, where TRPV1 mediated the expression of metalloproteinase-1 (MMP-1), MMP-2, MMP-3, MMP-9, and MMP-13 via a Ca^2+^-dependent protein kinase C-α (PKC-α) pathway (Li et al., 2007; Lee et al., 2008, 2009, 2011; Yoo et al., 2020). There is also evidence on the role of KC TRPV1 in regulating cell cycles (Radtke et al., 2011; Graham et al., 2013; Yoo et al., 2020). TRPV1 suppressed KC proliferation by promoting the degradation of epidermal growth factor receptor (EGFR) and inhibiting the downstream AKT/GSK3β/FoxO3a pathway (Yoo et al., 2020). Cell death of human KCs induced by heat at 42°C was relieved by a TRPV1 antagonist (Radtke et al., 2011). Cultured zebrafish KCs lost lamellipodia and demonstrated reduced motility when treated with TRPV1 antagonists (Graham et al., 2013).

TRPV3 is a sensor of a warm temperature (> 33-39°C) mainly expressed on KCs (Lee and Caterina, 2005; Moqrich et al., 2005). Activation of TRPV3 on KCs could excite sensory neurons by stimulating the release of Adenosine-5’-triphosphate (ATP) (Mandadi et al., 2009; Bang et al., 2010). Intraplantar injection of TRPV3 agonist triggered nociceptive behaviors in inflamed mice (Bang et al., 2010). Mice selectively overexpressing TRPV3 in K14-positive cells displayed augmented withdrawal behaviors to noxious heat only when TRPV1 was inhibited; this TRPV3-induced hyperalgesia is mediated by the production of COX-1 and PGE2 in KCs (Huang et al., 2008). Conversely, Huang et al. showed *trpv3* knockout mice, *trpv3/trpv4* double knockout mice, and wide-type mice had similar thermal sensitivity in both normal and complete Freund’s adjuvant (CFA)-induced inflammatory conditions, indicating the possible existence of redundant molecules that compensate for the functions of TRPV3 and TRPV4 (Huang et al., 2011). TRPV3 has also been identified on KCs in human skin biopsies. The expression of KCs TRPV3 was elevated in painful breast tissues, pruritic hypertrophic burn scars, and atopic dermatitis skin, but decreased in diabetic skin (Gopinath et al., 2005; Facer et al., 2007; Kim et al., 2016; Seo et al., 2020; Zhao et al., 2020). Additionally, TRPV3 promoted KC proliferation through an EGFR-dependent pathway (Wang et al., 2021).

TRPV4, which can be activated by a temperature between 25 and 34°C, extracellular hypotonicity, shear stress, or UVB, is also expressed on KCs (Chung et al., 2003; Lee and Caterina, 2005; Moore et al., 2013). When activated by UVB, TRPV4 upregulated the expression of endothelin-1 (ET-1) in KCs, which amplified TRPV4-dependent Ca^2+^ influx in a paracrine and/or autocrine manner (Moore et al., 2013). Topical application of a TRPV4 inhibitor or *Trpv4* ablation in K14-expressing cells attenuated UVB-induced nociceptive behaviors and tissue damage in mice (Moore et al., 2013). These findings demonstrated that KC TRPV4 mediated UVB-induced pain via ET-1 signaling. Activation of TRPV4 by intraplantar injection of its agonist also elicited acute inflammatory reactions and hypersensitivity to noxious mechanical stimuli (Bang et al., 2012). TRPV4 could be activated by histaminergic pruritogens to evoke itch behaviors by stimulating the phosphorylation of mitogen-activated protein kinase (MAPK) and extracellular signal-regulated kinase (ERK) in KCs (Chen et al., 2016).

TRPA1 is localized in a variety of tissues, including sensory neurons and epidermis (Atoyan et al., 2009). The functions of KCs TRPA1 are controversial. TRPA1 was reported to be activated by noxious cold (< 17°C) in human KCs *in vitro* (Nagata et al., 2005; Tsutsumi et al., 2010), whereas global *trpa1*-knockout mice and rats exhibited normal cold sensations (Bautista et al., 2006; Reese et al., 2020). In contrast, others found that *trpa1*-knockout mice showed behavioral deficits in response to cold (0°C) and mechanical stimuli (Kwan et al., 2006). Interestingly, although mouse KCs express little TRPA1 (Zappia et al., 2016; Sadler et al., 2020), mice with a selective deletion of *trpa1* in K14-expressing cells exhibited attenuated mechanosensitivity and decreased ATP release in both normal and CFA-induced inflammatory conditions (Zappia et al., 2016). The authors argued these effects might rely on other K14-expressing cells than adult KCs, such as Merkel cells (Zappia et al., 2016). TRPA1 can be activated by environmental irritants, and is involved in cutaneous inflammatory diseases, including allergic dermatitis, UVB-induced injury, and atopic dermatitis; but evidence specific to KCs TRPA1 was limited (Oh et al., 2013; Kang et al., 2017; Camponogara et al., 2020). The expression level of TRPA1 was very low in non-stimulated human KCs, but could be upregulated by TNF through nuclear factor κB (NFκB) and MAPK pathways (Luostarinen et al., 2021). TRPA1 in turn intensified inflammation by inducing the synthesis of monocyte chemoattractant protein 1 (MCP-1) and the production of IL-1α and IL-1β in KCs (Atoyan et al., 2009; Luostarinen et al., 2021). Topical application of a TRPA1 agonist induced the secretion of PGE_2_ and leukotriene B_4_ (LTB_4_) from human KCs in a pattern different from that induced by TRPV1 activation (Jain et al., 2011).

TRPM8 is a Ca^2+^-permeable ion channel located in the endoplasmic reticulum (ER) of human KCs (Bidaux et al., 2015). *In vitro* study showed, when activated by mild cold (24 to 33°C), epidermal TRPM8 coupled Ca^2+^ release from ER and Ca^2+^ uptake by mitochondria, which further modulated the synthesis of ATP and superoxide in mitochondria (Bidaux et al., 2015). In this way, epidermal TRPM8 regulated the proliferation and differentiation of KCs in a temperature-dependent manner (Bidaux et al., 2015).

In addition to the TRP family, KCs also express a temperature-sensitive ER transmembrane Ca^2+^ release-activated Ca^2+^ (CRAC) channel named STIM1, which is coupled to a plasma membrane pore-forming subunit named Orai1 (Figure 2) (Liu et al., 2019). When cultured KCs are heated to 42°C (heat-on) or cooled down to 25°C (heat-off), STIM1 is activated, and Orai1 mediates a Ca^2+^ influx (Liu et al., 2019). K14-positive cell-specific (but not neuron-specific) *stim1* knockout mice showed reduced warm-responding DRG neurons and altered temperature preference behaviors. However, both DRG and behavioral responsiveness to noxious heat remained unchanged in this mouse line. The above evidence suggested the role of KC STIM1/Orai1 in normal thermal sensation, but not noxious heat sensation (Liu et al., 2019).

### Voltage-gated cation channels

Voltage-gated sodium channel (VGSC, Nav) is widely distributed in neurons and KCs (Figure 2) (Wang et al., 2017). Ten subtypes of VGSC have been discovered to date, named Nav1.1-Nav1.9 and Nav X. Nav1.1, Nav1.6, and Nav1.8 were responsible for the release of ATP from rat KCs (Wijaya et al., 2020a). Compared with control skin biopsies, painful skin from patients with CRPS or post-herpetic neuralgia (PHN) exhibited an enhanced expression of Nav1.5, Nav1.6, Nav1.7, and an additional expression of Nav1.1, Nav1.2, and Nav1.8 (Wijaya et al., 2020a). A higher expression of Nav 1.7 was also detected in patients with SFN (Karl et al., 2021). These studies showed KCs VGSCs participate in neuropathic pain by promoting ATP release. VGSC also mediated ciguatoxin-induced activation and internalization of protease-activated receptor-2 (PAR2), leading to an increase in intracellular Ca2+ concentration [(Ca2+)i] in KCs (L’Herondelle et al., 2021). This finding provided an insight into the mechanisms of ciguatoxin-induced cutaneous sensory disturbance (L’Herondelle et al., 2021).

In addition to VGSC, L-type voltage-gated calcium channel (VGCC) subunit α1C has been found to be present in mouse and human KCs (Figure 2). The activation of L-type VGCC stimulates Ca^2+^ influx and delays the skin barrier recovery (Denda et al., 2006).

### Adrenoceptors

Adrenoceptors (AR) are activated by catecholamines released by the sympathetic nervous system during injury or stress (Wijaya et al., 2020a). Although α1- and β2-ARs are mainly expressed in smooth muscles, growing evidence has shown their expression in KCs (Li et al., 2013; Wijaya et al., 2020a). Expression of α1-AR was increased on KCs and nerve fibers around the injury site after a chronic constriction injury (CCI) or burn (Drummond et al., 2014a,^2015^). Interestingly, in a distal tibia fracture and cast immobilization model of CRPS I, the upregulation of KC α1-AR was also observed in the plantar skin of the contralateral limb (Drummond et al., 2014b). Moreover, a systemic, but not intraplantar, administration of an α1-AR antagonist relieved pain behaviors, suggesting the α1-AR upregulation is mediated by a central, but not a peripheral mechanism (Drummond et al., 2014b).

KCs ARs reinforce inflammation and pain sensitization by forming a positive feedback loop with pro-inflammatory cytokines. Application of TNF-α increased expression of the α1-AR subtype B in cultured KCs (Wijaya et al., 2020a,b). α1-AR activation further amplified the release of IL-6 from KCs (Drummond, 2014; Wijaya et al., 2020a). Similarly, KC β2-AR activation induced upregulation of IL-6 expression, along with the phosphorylation of MAPK, ERK, and c-Jun N-terminal kinase (JNK) (Li et al., 2013).

### Acetylcholine receptor

Acetylcholine (ACh) is a main neurotransmitter of the cholinergic system that acts through muscarinic and nicotinic receptors (mAChRs and nAChRs) (Zoli et al., 2018). There are five subtypes of mAChRs named M1-M5, and all of them are expressed in human KCs (Grando, 2012). Mammalian nAChRs are pentamers composed of 12 subunits named α2 to α10 and β2-β4 (Zoli et al., 2018). To date, α3, α5, α6, α7, α9, α10, β1, β2, and β4 subunits have been identified in human KCs (Grando, 1997; Nguyen et al., 2004; Zoli et al., 2018). The mAChR is a member of GCRPs that inhibits Ca^2+^ influx, while ionotropic nAChR stimulates Ca^2+^ influx (Grando, 1997). Human KCs are capable of synthesizing, secreting, and degrading ACh, indicating ACh works in a paracrine and/or autocrine manner in the epidermis (Grando et al., 1993). Previous studies have shown KCs AChRs mediated cell migration, differentiation, proliferation, adhesion, and apoptosis, and were responsible for wound healing and skin barrier recovery (Nguyen et al., 2000, 2003, 2004; Zia et al., 2000; Chernyavsky et al., 2004; Curtis et al., 2012; Ockenga et al., 2014; Sloniecka et al., 2015; Uberti et al., 2017, 2018). However, the roles of KCs AChRs in neurotransmission have not been illustrated. Topical administration of M1 receptor antagonist reversed mechanical allodynia and thermal hypoalgesia in a mouse model of DPN, whereas this study did not demonstrate whether these neuroprotective effects were attributed to KCs M1 receptors (Jolivalt et al., 2020).

### Glutamate receptor

As an important excitatory neurotransmitter, glutamate is a target for fast-acting ionotropic (iGluRs) and slow-acting metabotropic receptors (mGluRs) (Pereira and Goudet, 2018). Three major classes of iGluR have been discovered and named after their preferred ligands: N-methyl-D-aspartate (NMDA), α-amino-3-hydroxy-5-methyl-4-isoxazolole propionate (AMPA), and kainate acid (KA). KCs express NMDA receptors, AMPA receptors, and mGluRs (Genever et al., 1999; Cabanero et al., 2016; Xu et al., 2020). Intraplantar injection of NMDA, AMPA, or KA induced nociceptive behaviors in rats, which were alleviated by their antagonists (Zhou et al., 1996; Davidson et al., 1997). However, these studies did not elucidate whether the agents acted at their receptors in KCs or in FNEs.

N-methyl-D-aspartate (NMDA) receptors are heterotetramers consisting of two obligatory GluN1 subunits in combination with two GluN2 and/or GluN3 subunits (Vyklicky et al., 2014). GluN2B and subunit 2D have been identified in human KCs (Fischer et al., 2006; Xu et al., 2020). Using a rat chronic post-ischemia pain model of CRPS II, we reported upregulation and phosphorylation of GluN2B in KCs, which were associated with nociceptive behaviors (Xu et al., 2020). Moreover, GluN2B mediated activation of ERK and NF-κB in epidermis and DRGs, activation of astrocyte and microglia in spinal cord dorsal horns to initiate and maintain nociceptive behaviors, indicating the important role of KCs GluN2B in peripheral and central sensitization (Xu et al., 2020). The activation of NMDA receptors triggers Ca^2+^ influx, which regulates cell cycles of KCs (Genever et al., 1999; Fischer et al., 2004). NMDA receptors were found to be colocalized with a glutamate receptor, excitatory amino acid carrier type 1 (EAAC1) in stratum basale, and their distributions were significantly altered during wound re-epithelialization (Genever et al., 1999). Inhibition of KCs NMDA receptors led to a decrease in cell proliferation and differentiation, and an increase in apoptosis (Fischer et al., 2004; Morhenn et al., 2004). After barrier disruption with tape stripping, topical application of an NMDA receptor agonist or antagonist delayed or accelerated the barrier repair in hairless mice, respectively (Fuziwara et al., 2003).

α-amino-3-hydroxy-5-methyl-4-isoxazolole propionate (AMPA) receptors are tetramers composed of dimers assembled from 4 possible subunits, including GluA1-GluA4 (Cabanero et al., 2016). GluA4 has been found in human and mouse KCs (Cabanero et al., 2016). Ipsilateral skin biopsies exhibited a significantly lower KC GluA4 expression compared with contralateral skin from patients with PHN (Cabanero et al., 2016).

### γ-aminobutyric acid receptor

γ-Aminobutyric acid (GABA) is a main inhibitory neurotransmitter that acts on ionotropic (GABA_*A*_ or GABA_*C*_) and metabotropic (GABA_*B*_) receptors (Jiang et al., 2012). Ionotropic receptors are ligand-gated Cl^–^ channels that mediate fast GABA responses, while metabotropic receptors are G-protein-coupled receptors (GPCRs) that mediate slow responses (Jiang et al., 2012).

Cultured human KCs express functional GABA receptors that are able to induce an increase in intracellular Cl^–^ concentration, which was blocked by a GABA_*A*_ receptor antagonist (Denda et al., 2002). GABA_*A*_ receptors have also been identified in hairless mouse epidermis, where they mediate epidermal hyperplasia and skin barrier recovery after disruption (Denda et al., 2002).

GABA_*B*_ receptors are heterodimers containing GABA_*B1*_ and GABA_*B2*_ subunits (Jiang et al., 2012). When formalin was injected subcutaneously in mouse hindpaws to evoke pain, a co-localization of GABA_*B1*_ and GABA_*B2*_ was found on KCs and FNEs in the stratum spinosum around the injection site (Whitehead et al., 2012). The activation of KC GABA_*B*_ receptors elicits antinociceptive effects. Intraplantar injection of GABA_*B*_ receptor agonist alleviated PGE_2_-injection-induced allodynia (Whitehead et al., 2012). Application of GABA caused a decreased release of IL1-α and nerve growth factor (NGF) from cultured human KCs in neuroinflammatory conditions (Scandolera et al., 2018).

## Neurotransmission between keratinocytes and sensory neurons

The expression of neurotransmitter receptors on KCs and the *en passant* synaptic-like contacts between KCs and FNEs imply the possible signal transmission between them. Keratinocyte-sensory neuron coculture study and use of opto- or chemogenetic techniques to exclusively activate KCs have shed new light on the interaction between KCs and sensory neurons or nerve fibers (Talagas et al., 2020a).

### The ability of keratinocytes to excite sensory neurons

*In vivo* studies on KCs on peripheral nociceptive transduction are limited by the small diameter of the peripheral nociceptive nerve endings and the difficult experimental accessibility (Klusch et al., 2013). Nevertheless, *in vitro* studies have shed some lights on the role of KCs in nociception. Using a compartmented chamber coculture combined with live-cell imaging and atomic force microscopy, Klusch et al. (2013) reported structurally nanoscaled close contact between porcine KCs and sensory nerve endings *in vitro*. They found functionally direct cross talk between KCs and neurites. Mechanically stimulated KCs caused a transient increase in calcium concentration in nearby neuritis (Klusch et al., 2013). In cocultures of human KCs and rat DRG sensory neurons, neuronal currents and conductances recorded by patch clamp significantly increased only after the application of capsaicin to the KCs contacting with neurons, indicating sensory neuron activation and AP generation subsequent to nociceptive activation of KCs (Talagas et al., 2020c). In a coculture system of KCs and mouse DRG neurons, mechanical stimuli evoked an increase in [Ca^2+^]_*i*_ in KCs, followed by an [Ca^2+^]_*i*_ increase in adjacent DRG neurons after a time lag, indicating DRG neurons might be excited by KCs (Koizumi et al., 2004). Similarly, mechanical stimulation of a single KC cell induced a propagation of [Ca^2+^]_*i*_ increase in neighboring KCs and rat DRG neurons (Tsutsumi et al., 2011; Shindo et al., 2021). This phenomenon was observed when they were seeded together at the same time, but not when KCs were seeded first and then DRG neurons were seeded on KCs (Tsutsumi et al., 2011). Exposure to the odorant chemicals induced [Ca^2+^]_*i*_ increase in monocultured KCs and TG neurons cocultured with KCs, but not in monocultured TG neurons (Sondersorg et al., 2014). These findings suggest the necessity of contacts between KCs and sensory neurons in neurotransmission.

Cell-specific optogenetic approaches have allowed researchers to study the specific roles of KCs *in vivo*. In a mouse line that selectively expresses a light-sensitive cation channel (channelrhodopsin-2) in K14-expressing cells, exposure to light could depolarize and activate KCs (Baumbauer et al., 2015; Moehring et al., 2018). In contrast, when a light-driven inward chloride pump (halorhodopsin) or outward proton pump (archaerhodopsin-3, Arch) was exclusively expressed in K14-expressing cells, KCs could be hyperpolarized and inhibited by light (Baumbauer et al., 2015; Moehring et al., 2018; Sadler et al., 2020). The optogenetic activation of KCs was sufficient to trigger action potentials in Aδ- and C-fibers, and initiated nociceptive behaviors (Baumbauer et al., 2015; Moehring et al., 2018). Conversely, an optogenetic silence of KCs reduced action potentials of nociceptors, as well as behavioral responses to both noxious and innoxious mechanical stimuli (Baumbauer et al., 2015; Moehring et al., 2018). Furthermore, an optogenetic inhibition of KCs also attenuated normal cold and heat sensation (Sadler et al., 2020).

Keratinocytes (KCs) were also associated with the hyperexcitability of sensory neurons in neuropathic conditions. In a rat model of sciatic nerve neuroma, human KCs were transplanted into the ligated and transected sciatic nerve; they assembled in a structure resembling stratum spinosum. The transplanted rats displayed spontaneous pain behaviors, and their DRG neurons fired spontaneously at resting potential (Radtke et al., 2010).

### Adenosine-5′-triphosphate signaling

Adenosine-5′-triphosphate (ATP) is a key extracellular signaling molecule participating in neurotransmission and neuromodulation (Suurvali et al., 2017). ATP can bind to and activate two main classes of purinergic cell surface receptors: ionotropic P2X receptors and metabotropic P2Y receptors (Suurvali et al., 2017). The fast-acting P2X receptors are ligand-gated cation channels, depolarizing the cell membrane upon activation (Alves et al., 2018). The slow-acting P2Y receptors are GPCRs, regulating the release of Ca^2+^ from an intracellular store (Alves et al., 2018).

**FIGURE 3:**
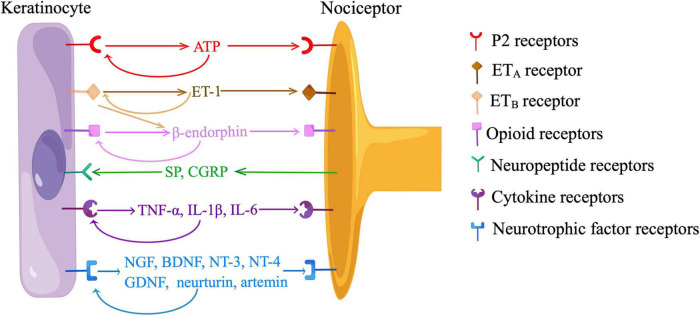
Communications between keratinocytes and nociceptors. ATP, adenosine-5’-triphosphate; ET-1, endothelin-1; ET_A_, endothelin-1 receptor A; ET_B_, endothelin-1 receptor B; SP, substance P; CGRP, calcitonin gene-related peptide; TNF-α, tumor necrosis factor-α; IL, interleukin; NGF, nerve growth factor; BDNF, brain-derived neurotrophic factor; NT, neurotrophin; GDNF, glia-derived neurotrophic factor. Neurotransmission between keratinocytes and sensory neurons is dependent on the release of ATP from keratinocytes and the activation of P2 receptors on nociceptors. Keratinocytes produce ET-1, which binds to ET_A_ on nociceptors to evoke pain. ET-1 also activates ET_B_ on keratinocytes, stimulating the release of β-endorphin, which binds to opioid receptors on nociceptors to relieve pain. Keratinocytes participate in neuroinflammation by producing neurotrophic factors and pro-inflammatory cytokines in response to neuropeptides released from nociceptors. Keratinocytes also express receptors of ATP, β-endorphin, neurotrophic factors, and pro-inflammatory cytokines, indicating the existence of paracrine and/or autocrine mechanisms. (By Figdraw).

Recent *in vitro* and *in vivo* data have indicated neurotransmission between KCs and sensory neurons depended on the release of ATP from KCs and the activation of P2 receptors on sensory neurons (Figure 3). Cultured human KCs constitutively released ATP at a low level (Dixon et al., 1999). Application of a depolarizing stimulus (10mM K^+^) led to a significant increase in ATP release over baseline levels in cultured KCs (Zhao et al., 2008). When KCs were cultured with HEK293 cells expressing P2X2 receptors (sniffer cells), sniffer cells exhibited inward currents when an adjacent KC was mechanically stimulated, and the current was abolished by an ATP-degrading enzyme, apyrase (Moehring et al., 2018). In a coculture model of sensory neurons and KCs, ATP was released from the mechanically or chemically stimulated KCs, and ATP levels were also elevated on the neuron cell surface closer to the stimulated KCs (Koizumi et al., 2004; Sondersorg et al., 2014; Shindo et al., 2021). KC-initiated propagation of Ca^2+^ wave in the coculture was at least partially blocked by apyrase or a P2 receptor antagonist (Tsutsumi et al., 2011; Sondersorg et al., 2014; Sadler et al., 2020). The amount of ATP release correlated with the intensity of cutaneous mechanical stimuli in glabrous hindpaw skin of mice (Moehring et al., 2018). Subcutaneous injection of apyrase decreased mechanical responsiveness and action potentials fired from nociceptors in naive mice, and also abolished behavioral responses evoked by optogenetic activation of KCs (Moehring et al., 2018). However, the treatment of apyrase did not produce additional effects during optogenetic inhibition of KCs, suggesting the KCs is a major source of ATP (Moehring et al., 2018). Furthermore, behavioral responses were reduced either by a subcutaneous injection of a P2X4 receptor inhibitor or by a neuron-specific deletion of *p2x4*, indicating ATP acts through P2X4 receptors on sensory neurons (Moehring et al., 2018; Sadler et al., 2020). Additionally, both KCs and FNEs expressed ecto-5’-nucleotidase (NT5E), a membrane-anchored protein hydrolyzing adenosine 5’-monophosphate (AMP) to adenosine (Sowa et al., 2010). Inflammation or nerve injury induced nociceptive behaviors were intensified in *Nt5e^–/–^* mice, implying the role of purinergic signaling in cutaneous nociception (Sowa et al., 2010).

Multiple subtypes of P2 receptors are expressed on KCs. In rat epidermis, P2Y1 and P2Y2 receptors are expressed in stratum basale, controlling cell proliferation; P2X5 receptors are predominately expressed in stratum basale and stratum spinosum, where they promote differentiation; P2X7 regulates terminal differentiation and death of KCs in stratum corneum (Greig et al., 2003a,b; Dussor et al., 2009). P2X1, P2X2, P2X3, P2X7, P2Y4, and P2Y6 receptors have also been identified in KCs (Burrell et al., 2003; Inoue et al., 2005). Exogenous application of ATP produced an increase in [Ca^2+^]_*i*_, and enhanced the expression of P2X receptors in KCs, indicating a possible paracrine and/or autocrine of ATP (Koizumi et al., 2004; Inoue et al., 2005). ATP release and the expression of P2X and P2Y receptors were also increased in response to inflammation, UVB irradiation, and skin injury in KCs, possibly resulting in increased nociceptor sensitivity (Greig et al., 2003a; Inoue et al., 2005; Dussor et al., 2009).

### Endogenous opioid system

The endogenous opioid system consists of neuropeptides (endorphins, enkephalins, endomorphins, and dynorphins) and opioid receptors (ORs, μ, δ, and κ) (Cirillo, 2021). δ-OR, κ-OR, μ-OR, and nociceptin/orphanin FQ peptide receptor (NOP) are expressed on KCs (Bigliardi et al., 1998, 2015; Andoh et al., 2004; Tominaga et al., 2007). KCs are able to secrete neuropeptide precursor forms, including proenkephalin (PENK), proopiomelanocortin (POMC), and prodynorphin, as well as their mature proteins (Bigliardi et al., 2016).

β-endorphin, a POMC-derived neuropeptide plays an important role in the endogenous analgesic circuit. Immunohistochemistry showed KCs clustered around unmyelinated FNEs were positive for β-endorphin, suggesting KCs can communicate with FNEs by secreting β-endorphin (Bigliardi-Qi et al., 2004). An elevated level of circulating β-endorphin was associated with increased nociceptive thresholds in mice (Fell et al., 2014). The analgesic effects were abolished by a selective silence of p53-mediated POMC induction in K14-positive cells, indicating β-endorphin was mainly produced by KCs (Fell et al., 2014). β-endorphin-induced antinociception was also inhibited by the functional deletion of μ-OR or G-protein-coupled inwardly rectifying potassium channel 2 (GIRK2), or a subcutaneous injection of a κ-OR antagonist, suggesting β-endorphin acts at μ and κ-ORs on nociceptors and inhibits membrane excitability by activating GIRK (Khodorova et al., 2003; Ibrahim et al., 2005).

β-endorphin signaling was modulated by cell membrane receptors on KCs, such as cannabinoid receptor 2 (CB2) and ET-1 receptor B (ETB) (Khodorova et al., 2003; Ibrahim et al., 2005; Katsuyama et al., 2013; Gao et al., 2016). The activation of CB2 receptor stimulated the release of β-endorphin from KCs through the Gi/o-Gβγ-MAPK-Ca^2+^ pathway, and attenuated nociceptive behaviors in rodent models of inflammatory pain and capsaicin-induced pain (Ibrahim et al., 2005; Katsuyama et al., 2013; Gao et al., 2016). KCs secreted ET-1, a pain mediator that acts in a paracrine and/or autocrine manner by binding to ET_*A*_ and ET_*B*_ on KCs and FNEs (Figure 3) (Khodorova et al., 2003). The activation of ET_*A*_ on FNEs evoked nociceptive behaviors, which were alleviated by the activation of ETB in KCs (Khodorova et al., 2003). This ET_*B*_-dependent analgesia was blocked by a subcutaneous injection of antiserum to β-endorphin, and ETB and β-endorphin were co-localized in KCs adjacent to FNEs (Khodorova et al., 2003). These findings, along with *in vitro* data in cultured KCs, showed ET_*B*_ mediates the release of β-endorphin from KCs (Figure 3).

## Roles of keratinocytes in neuroinflammation

“Neuroinflammation” refers to a mechanism by which sensory nerves contribute to inflammation (Choi and Di Nardo, 2018). Classically, KCs participate in neuroinflammation by producing NGF, glia-derived neurotrophic factor (GDNF), and pro-inflammatory cytokines in response to neuropeptides released from FNEs. These mediators in turn stimulate FNEs and immune cells, creating a bidirectional feedback loop that amplifies inflammation and contributes to peripheral sensitization (Figure 3) (Choi and Di Nardo, 2018).

### Stimulation of keratinocytes by neuropeptides

Intraepidermal nerve fibers can be classified as peptidergic and nonpeptidergic fibers, which terminate in the stratum spinosum and granulosum, respectively, providing an opportunity of layer-specific nociceptive transduction (Zylka et al., 2005; Basbaum et al., 2009). Peptidergic fibers release either substance P (SP) and/or calcitonin gene-related peptide (CGRP) and respond to NGF, while nonpeptidergic fibers are sensitive to GDNF (Basbaum et al., 2009). As a member of the tachykinin family, SP acts on neurokinin 1 (NK1) receptors with a high affinity, as well as NK2 and NK3 receptors with a relatively low affinity (Steinhoff et al., 2014). There are two distinctive forms of CGRP: α-CGRP and β-CGRP (Hou et al., 2011). The CGRP receptor complex consists of a ligand-binding GPCR named calcitonin-like receptor (CLR) and two accessory proteins, receptor activity modifying protein 1 (RAMP1) and CGRP-receptor component protein (RCP) (Hou et al., 2011).

Previous studies have indicated human KCs express NK1 receptor, CLR, RAMP1, and RCP, while mouse KCs additionally express NK2 receptors (Song et al., 2000; Liu et al., 2006; Hou et al., 2011). Human KCs were able to release endogenous SP when stimulated by exogenous SP (Bae et al., 1999). A strong and widespread expression of CGRP (predominantly β-CGRP) was discovered on KCs in the skin biopsies of patients with PHN or CRPS I, monkeys infected with simian immunodeficiency virus, and rat models of spinal nerve ligation, CCI, or CFA-induced inflammation (Hou et al., 2011). Exposure to SP and CGRP induced an increased expression of these peptides and an upregulation of their receptors in KCs, indicating a paracrine and/or autocrine mechanism (Paus et al., 1995; Hou et al., 2011; Shi et al., 2013). SP and CGRP could also promote the proliferation and nitric oxide (NO) production of KCs (Paus et al., 1995; Yaping et al., 2006; Roggenkamp et al., 2013; Shi et al., 2013).

Substance P (SP) and/or CGPR induced an enhanced production of NGF and pro-inflammatory cytokines as well as an upregulation of IL-1 receptor on KCs (Li et al., 2009; Shi et al., 2011, 2013; Wei et al., 2012). These effects are mediated by NK1 and CLR receptors, both of which regulate the phosphorylation of p38 and ERK (Shi et al., 2013). NK1 could also activate JNK, cathepsin B, caspase-1, and Nacht, leucine-rich repeat and pyrin domain containing protein (NALP1) inflammasome (Li et al., 2009; Shi et al., 2011, 2013). In a tibia fracture and immobilization rat model of CRPS I, an increased expression of SP and CGPR was observed in the sciatic nerve, while KCs in hindpaw skin were activated and underwent rapid proliferation with an enhanced expression of NK1 (Li et al., 2009, 2010; Wei et al., 2009a,^2012^, ^2016^; Kingery, 2010; Guo et al., 2014). Fracture and/or immobilization induced mechanical allodynia, unweighting, warmth, and edema, in associations with an elevated release of TNF-α, IL-1β, IL-6, and NGF from KCs (Li et al., 2009, 2010; Wei et al., 2009a,b; Whitehead et al., 2012; Kingery, 2010; Guo et al., 2012, 2014). These effects were also created by an intraplantar injection of SP in normal rats, and were attenuated in SP or CGPR receptor deficient mice, or by systemic treatments of NK1, IL-1, or IL-6 receptor antagonists, or anti-NGF antibody (Li et al., 2009; Wei et al., 2009a,^2012^, ^2016^; Kingery, 2010; Guo et al., 2012, 2014). Skin biopsies and blisters from patients with acute CRPS displayed an elevated expression of TNF-α and IL-6 in KCs (Huygen et al., 2004; Birklein et al., 2014). In addition to CRPS, a proliferation of KCs and an increased release of IL-1β, IL-6, and TNFα were observed in a murine incisional model for postsurgical pain (Clark et al., 2007; Guo et al., 2020). These changes were more persistent in diabetic mice, in associations with a prolonged postoperative pain hypersensitivity (Guo et al., 2020).

In addition to SP and CGPR, other neuropeptides, such as neurokinin A, vasoactive intestinal polypeptide (VIP), and galanin (GAL), have similar effects in stimulating the release of pro-inflammatory mediators from KCs (Burbach et al., 2001; Dallos et al., 2006).

### Release of neurotrophic factors from keratinocytes

NGF is a main neurotrophic factor participating in neuroinflammation. As a member of the family of neurotrophins, NGF regulates the survival and differentiation of peripheral sensory and sympathetic neurons, and the synthesis of neuropeptides and neurotransmitters (Yaping et al., 2006). NGF acts on a low-affinity p75 receptor and a high-affinity tropomyosin-related kinase receptor A (TrkA) (Pincelli and Marconi, 2000).

Keratinocytes (KCs) is a major source of NGF in the epidermis (Di Marco et al., 1991; Pincelli et al., 1994; Pincelli and Marconi, 2000). In addition to neuropeptides, TNF-α and histamine could stimulate the production of NGF from KCs via the Raf/MEK/ERK pathway (Woolf et al., 1997; Takaoka et al., 2009; Wijaya et al., 2020a,b). Human KCs expressed both p75 and TrkA receptors, suggesting the existence of an autocrine and/or paracrine system (Pincelli et al., 1994; Pincelli and Marconi, 2000). TrKA receptors were primarily located in stratum basale, where they stimulated proliferation and prevented apoptosis of KCs upon activation (Pincelli et al., 1994; Bronzetti et al., 1995; Marconi et al., 1999; Pincelli and Marconi, 2000). Conversely, p75 has a pro-apoptotic role in KCs (Truzzi et al., 2011). In a coculture model of DRG neurons and KCs, NGF level was enhanced as compared to the monocultured neurons or KCs, leading to a stimulation of axonal outgrowth and KC proliferation (Ulmann et al., 2007; Radtke et al., 2013).

Keratinocytes (KCs)-derived NGF was able to increase epidermal innervation and the excitability of sensory neurons, KCs were thus involved in a variety of neuropathic conditions (Barker et al., 2020). CCI induced an increased production of the precursor form of NGF (proNGF) in KCs in rats (Peleshok and Ribeiro-da-Silva, 2012). Intraplantar injection of CFA triggered a long-last upregulation of NGF and p75 expression in KCs, which led to an upregulation of CGRP in primary sensory neurons (Woolf et al., 1997; Sivilia et al., 2008). Blockade of p75 by a neutralizing antibody or decomposition of proNGF by plasmin ameliorated CFA-induced inflammatory thermal hyperalgesia in rats (Watanabe et al., 2008). Muscle incision caused an increased production of NGF in KCs in rats; and pretreatments of anti-NGF antibody decreased incision-induced hyperalgesia behaviors (Wu et al., 2009). Transgenic mice that selectively overexpress NGF in K14-positive cells displayed a hypertrophy of the peripheral nervous system and a hyperalgesia to noxious mechanical stimuli, while mice with NGF antisense in KCs displayed hypotrophy (Davis et al., 1993; Albers et al., 1994). A subcutaneous or intradermal injection of NGF evoked long-lasting local hyperalgesia in healthy humans (Barker et al., 2020). Patients with diabetic polyneuropathy or leprosy exhibited a decreased level of NGF in skin and a compensatory increased TrKA expression in KCs, which contributed to hypoalgesia (Anand et al., 1996; Terenghi et al., 1997; Facer et al., 2000).

In addition to NGF, KCs could produce other neurotrophin family members, including brain-derived neurotrophic factor (BDNF), neurotrophin-3 (NT-3), and neurotrophin-4 (NT-4), as well as GDNF family members, including GDNF, neurturin and artemin (Botchkarev et al., 1999; Malin et al., 2006; Wang et al., 2013). Expression of artemin was enhanced after intraplantar injection of CFA (Malin et al., 2006; Ikeda-Miyagawa et al., 2015). Selective overexpression of BDNF, neurturin, or artemin in K14-positive cells induced an increased behavioral sensitivity and nociceptor responses to mechanical, thermal, and cold stimuli (Albers et al., 2006; Elitt et al., 2006; Wang et al., 2013; Jankowski et al., 2017).

## Conclusion and prospects

In summary, recent literature has indicated that KCs are not just the block builder in the skin but also able to sense and transduce noxious and innoxious stimuli to sensory neurons. KCs express a variety of receptors for ion channels and neurotransmitters. At this time, only TRPV1 on KCs has been implicated in nociceptive transduction. Other ion channels/receptors seem to contribute to more regulation than transduction of nociceptive signals. KCs serve as both sources and targets of neuroactive and inflammatory mediators. The emerging roles of KCs in nociception has extended the knowledge in peripheral sensitization of pain. Understanding of the pivotal roles of KCs could provide a solid foundation for targeting KCs to treat pain.

## Author contributions

JX: conceptualization. XX and LX: literature review and analysis. XX: writing – original draft preparation. CY, LX, and JX: writing – review and editing. LX and JX: supervision and funding acquisition. All authors contributed to manuscript revision, read, and approved the submitted version.
